# Exploring the impact of biological alterations in the superior thalamic radiations on exploratory eye movements in attenuated psychosis syndrome

**DOI:** 10.3389/fpsyt.2024.1323786

**Published:** 2024-06-13

**Authors:** Yu Arai, Naoyuki Katagiri, Hiromi Tagata, Takashi Uchino, Junichi Saito, Yusuke Shido, Kouhei Kamiya, Masaaki Hori, Masafumi Mizuno, Takahiro Nemoto

**Affiliations:** ^1^ Department of Neuropsychiatry, Toho University Graduate School of Medicine, Tokyo, Japan; ^2^ Department of Neuropsychiatry, Toho University Faculty of Medicine, Tokyo, Japan; ^3^ Department of Neuropsychiatry, Saiseikai Yokohamashi Tobu Hospital, Yokohama, Japan; ^4^ Department of Psychiatry and Implementation Science, Toho University Faculty of Medicine, Tokyo, Japan; ^5^ Department of Radiology, Toho University Omori Medical Center, Tokyo, Japan; ^6^ Tokyo Metropolitan Matsuzawa Hospital, Tokyo, Japan

**Keywords:** attenuated psychosis syndrome, superior thalamic radiation, fixations, scan path lengths, exploratory eye movement, diffusion tensor imaging, TractSeg

## Abstract

**Introduction:**

Aberrant fixation and scan paths in visual searches have been repeatedly reported in schizophrenia. The frontal eye fields (FEF) and thalamus may be responsible for fixation and scan paths. These two regions are connected by superior thalamic radiation (STR) in humans. Studies have reported reduced fixation numbers and shortened scan path lengths in individuals with attenuated psychosis syndrome (APS) and schizophrenia. In this study, we hypothesized that STRs in the white matter fiber bundles of impairments underlie abnormalities in fixation and scan path length in individuals with APS.

**Methods:**

Twenty-one individuals with APS and 30 healthy controls participated in this study. All participants underwent diffusion tensor imaging, and fractional anisotropy (FA) values of the left and right STR were analyzed using the novel method TractSeg. The number of eye fixations (NEF), total eye scanning length (TESL), and mean eye scanning length (MESL), derived using the exploratory eye movement (EEM) test, were adopted to evaluate the fixation and scan path length. We compared the FA values of the bilateral STR and EEM parameters between the APS and healthy control groups. We investigated the correlation between bilateral STR and EEM parameters in the APS and healthy control groups.

**Results:**

NEF, TESL, MESL, and the FA values of the left STR were significantly reduced in individuals with APS compared to healthy controls. The left STR FA value in the APS group was significantly positively correlated with the MESL (*r* = 0.567, *p* = 0.007). In addition, the right STR FA value of the APS group was significantly correlated with the TESL (*r* = 0.587, *p* = 0.005) and MESL (*r* = 0.756, *p* = 0.7×10^-4^).

**Discussion:**

These results demonstrate that biological changes in the STR, which connects the thalamus and FEF, underlie abnormalities in fixation and scanning. Recently, aberrations in the thalamus–frontal connection have been shown to underlie the emergence of psychotic symptoms. STR impairment may be a part of the biological basis of APS in individuals with subthreshold psychotic symptoms.

## 1 Introduction

Approximately 80% of patients with schizophrenia and 66% of patients with the first episode before antipsychotic administration are reported to have genuine motor abnormalities (1, 2). These motor abnormalities and psychiatric symptoms observed in patients with schizophrenia may be caused by the disruption of neural networks in the brain. Therefore, investigating the biological background of motor abnormalities in schizophrenia may clarify part of the pathology of the aberrant neural networks in the brain underlying schizophrenia.

In 1908, Diefendorf and Dodge reported abnormal eye movements in schizophrenia (3). Abnormalities in saccades, smooth pursuit, and visual search have been identified as characteristics of eye movements in schizophrenia (4). Repeated short stops of eye movement between scanning objects are defined as fixations, and the set of fixations connected by saccades is defined as the scan path (4, 5). In studies on visual search in schizophrenia, a reduced number of fixations and shortened scan path lengths have been repeatedly reported by several studies (5–7). In the latest study in 336 patients with schizophrenia and 1,254 healthy controls, a significant reduction in the scan path length and number of fixations has been reported using the free viewing test (8). This reduction corroborates with the reduced number of fixations and shortened scan path lengths observed in patients with schizophrenia. Considering that the pathway connecting fixations comprises the scan path, impairment of the function of fixation is shown to provoke abnormalities in the scan path and fixation.

Fixation neurons in the frontal eye field (FEF) are responsible for maintaining fixation and discharge during fixation. Conversely, a reduction in the activity of fixation neurons within the FEF increases the activity of saccade-related movement neurons (9). Spontaneous eye movements are controlled by motor and fixation cells in the FEF (10), which play a critical role in the early stages of visual search (11, 12).

The localization of the FEF, which may include fixation neurons, varies depending on the differences in studies and methods. In general, the FEF is more widely distributed posteriorly in humans than in primates, including the precentral cortex (13–15). The frontal cortex is thought to have hierarchical anatomical and functional gradients along its rostral-to-caudal axis. The pathological changes in the lowest layer of the frontal cortex underlie the impairment of a broad range of the frontal cortex, including the prefrontal cortex, which is in the upper layer (16).

Several regions other than the frontal cortex are also responsible for fixation (17). Mounting evidence shows a relationship between fixation and the thalamus. Rafal et al. (2004) investigated patients with thalamic lesions and reported that the thalamus was involved in the control of fixation for visually triggered saccades (18–20). Furthermore, several studies reported a relationship between fixation abnormality and thalamic impairments in schizophrenia (19, 20). Fukumoto-Motoshita et al. (2009) proposed high activation of the thalamus in fixation tasks in schizophrenia (19). The thalamus has a neural connection with the cerebral cortex and the thalamocortical pathway. McAvoy et al. (2012) have reported a relationship between fixation and thalamocortical connectivity (21). Recently, converging evidence has corroborated that impairment of the thalamocortical pathway is central to the pathophysiology of schizophrenia (22, 23). The FEF that includes fixation neurons is distributed in the posterior part of the frontal cortex, including the precentral cortex in humans (13–15), and these regions have a neural connection with the thalamus via superior thalamic radiation (STR) (24, 25). Few studies have directly investigated the precise impairment of the STR in schizophrenia *in vivo* because of methodological difficulties. However, the STR is included in the corona radiata, and reduced fractional anisotropy (FA) values in the corona radiata among patients with schizophrenia have been reported using diffusion tensor imaging (DTI) (24, 26). FA values obtained through DTI are regarded as a measure of white matter fiber bundle integrity that facilitates communication between different brain regions. Additionally, in a longitudinal study of individuals at risk of developing psychosis (ARMS group), a noteworthy reduction in FA values within the left superior corona radiata was observed among those who eventually developed psychosis compared to those who did not (27). These findings imply that biological alterations within the corona radiata, including the STR, become evident in individuals at risk for psychosis and in those with established schizophrenia.

Attenuated psychosis syndrome (APS) affects 85% of ARMS individuals (28). Shido et al. investigated abnormal eye movements in individuals with APS using an exploratory eye movement (EEM) test. The EEM test comprises the following parameters: number of eye fixations (NEF), total eye scanning length (TESL), mean eye scanning length (MESL), and responsive search score (RSS), calculated from the point of gaze and movement distance of eye movements (29), all of which have been reported to be significantly reduced in schizophrenia compared to other mental disorders, such as depression and anxiety disorders (6). In this study, the NEF, TESL, and RSS were significantly lower in the APS group than in the healthy control group (30). Regarding fixation and scan path length, these results are in line with the reduction in total eye scanning length, that is, TESL reduction and significant NEF reduction, which were repeatedly reported in previous reports on EEM tests in schizophrenia (6, 7). Kojima et al. (2019) focused on the subjectivity disorder in schizophrenia and reported that EEM tasks, including comparison matching and reminder tasks, can assess the disorder in schizophrenia (31). Subjectivity disorder or self-disorder is the core feature of schizophrenia. Thus, there is a possibility that investigating the EEM disturbance clarifies the core pathophysiology of APS as well as schizophrenia. Meanwhile, due to the methodological difficulty in investigating the precise structure of the STR *in vivo*, whether the biological changes of the STR underlie the abnormality of visual search in the APS group remains unclear.

The advent of a new method, TractSeg, enables the analysis of white matter tract segmentation using a direct approach that provides complete and accurate segmentation of the entire brain, including the STR (32). In this study, we hypothesized that biological changes in the STR are associated with EEM abnormalities in individuals with APS. We used TractSeg to investigate the FA values in the STR and examined the differences in the FA values of the STR and EEM parameters (NEF, TESL, MESL, and RSS) between the APS and healthy control groups. Furthermore, we examined the relationship between changes in STR and EEM parameters in the APS and healthy control groups.

## 2 Materials and methods

### 2.1 Participants

All individuals who visited the Department of Psychiatry at Toho University Omori Medical Center underwent The Prevention Through Risk Identification, Management, and Education (PRIME) Screen-Revised (PS-R) program to screen potential individuals with APS. Subsequently, the Japanese versions of the Structured Interview for Prodromal Syndromes and the Scale of Prodromal Symptoms (SIPS/SOPS) were assessed to identify individuals with APS criteria (33–35). APS individuals were native Japanese of 16–40 years of age and had no history of alcohol dependence, substance abuse, or neurological illnesses. Healthy control participants were recruited from independent sources in the community and were interviewed in detail by experienced psychiatrists. Similar to the APS group, healthy controls were native Japanese of 16–40 years of age, and none had a history of alcohol dependence, substance abuse, or neurological illnesses. The Edinburgh Handedness Inventory was used to determine handedness in both the healthy controls and the APS group, and all analyses were performed with right-handed participants (36). The study procedure was explained, and written informed consent was obtained from all participants. For participants under 20 years of age, we explained the contents of this research to their parents or legal representatives and obtained their written informed consent. This study was performed in accordance with the Declaration of Helsinki of the World Medical Association and approved by the Ethics Committee of Toho University Omori Medical Center (A19078).

### 2.2 Exploratory eye movements

The dominant eye was identified using the Miles test Prior to testing exploratory eye movements (37). The participants were first asked to extend their arms out in front of them. Secondly, they were asked to create a triangle between their thumbs and forefingers by placing their hands together at a 45-degree angle. They were then asked to center this triangle on a wall clock with both eyes open. Finally, they were asked to close either the right or left eye. If the object stays centered, the opened eye is determined to be the dominant eye. If the object is no longer framed by their hands, the closed eye is considered the dominant eye. We acquired four EEM parameters, NEF, TESL, MESL, and RSS, using a digital eye-mark recording system (Nac Image Technology, EMR-NS, Tokyo, Japan). The device includes an eye camera that detects the corneal reflection of infrared light and a 15-inch LCD monitor that displays figures for EEM tasks to identify eye movements (38). A computer automatically recorded and analyzed the eye movements.

**Figure 1:**
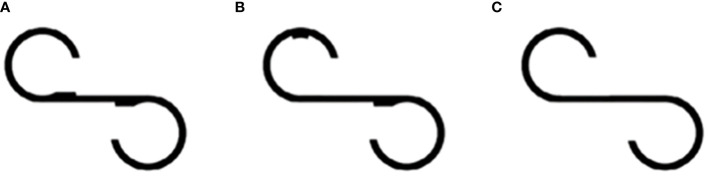
S-shaped figure. **(A)** Original target figure; **(B, C)** two figures are slightly different from the target.

First, the original S-shaped figure (**Figure 1A**) was displayed on the LCD monitor, and the participants were asked to observe it for 15 s. Subsequently, participants were asked to “Please draw the next figure after finishing this test,” and once again, the original S-shaped figure was displayed for an additional 15 s. Eye movements were continuously tracked while the participants gazed at the figure, and the fixation point of view was recorded when the eye movements stopped for more than 0.1 s at a specific location. The number of fixations of the participants was recorded as NEF, total eye scanning length as TESL, and mean eye scanning length as MESL.

For the comparison task, the participants were asked to look at a figure featuring a single bump located in a different position (**Figure 1B**) for 15 s. Following this observation period, participants were asked whether this figure differed from the original one, and if their response was affirmative, they were asked to specify the observed difference. Participants were also prompted to identify any additional discrepancies. Subsequently, the responsive search score (RSS) was automatically calculated based on the number of fixation locations within a 5-s window. The figure was divided into seven segments, and the maximum attainable RSS score for this task was seven. The same procedure was repeated using another figure, which was identical to the original (**Figure 1A**). Participants were expected to report no differences.

Subsequently, a comparison task was performed using a figure without bumps (**Figure 1C**). Similar to the initial comparison task, eye movements were automatically recorded after the posing question, and the RSS was calculated. The maximum RSS for the entire test was 14. Finally, the participants were asked to draw a target figure on the paper. Participants with NEF below 11 or whose RSS was 0 were excluded from the analysis, as their responses were considered unreliable for accurate measurements.

As a sample, we show the results of an EEM performed by a control subject (**Supplementary Figure 1**).

### 2.3 Acquisition conditions for magnetic resonance imaging

Magnetic Resonance Imaging (MRI) data were obtained using a 1.5-T scanner (Signa HDxt, GE Medical Systems, Waukesha, WI, USA) with a single-shot, spin-echo echo-planar imaging sequence. Diffusion MRI data were acquired with b = 1000 s/mm^2^ along 30 noncollinear directions and a single b = 0 s/mm^2^ volume. The other scan parameters included TE = 77 ms, TR = 13000 ms, 3 mm^3^ isotropic voxel, FOV = 240 × 240 mm, and 60 slices.

### 2.4 Image processing

Raw images were denoised and corrected for Gibbs ringing. Eddy currents and motion corrections were performed using the eddy tool in the FMRIB Software Library (FSL Version 6.0.4). Finally, the images were minimally smoothed using a Gaussian kernel with sigma = 1 mm to suppress the effects of residual noise and Gibbs artifacts. The DTI FA map was computed using a standard weighted least-squares fit implemented in MRTrix3.

**Figure 2:**
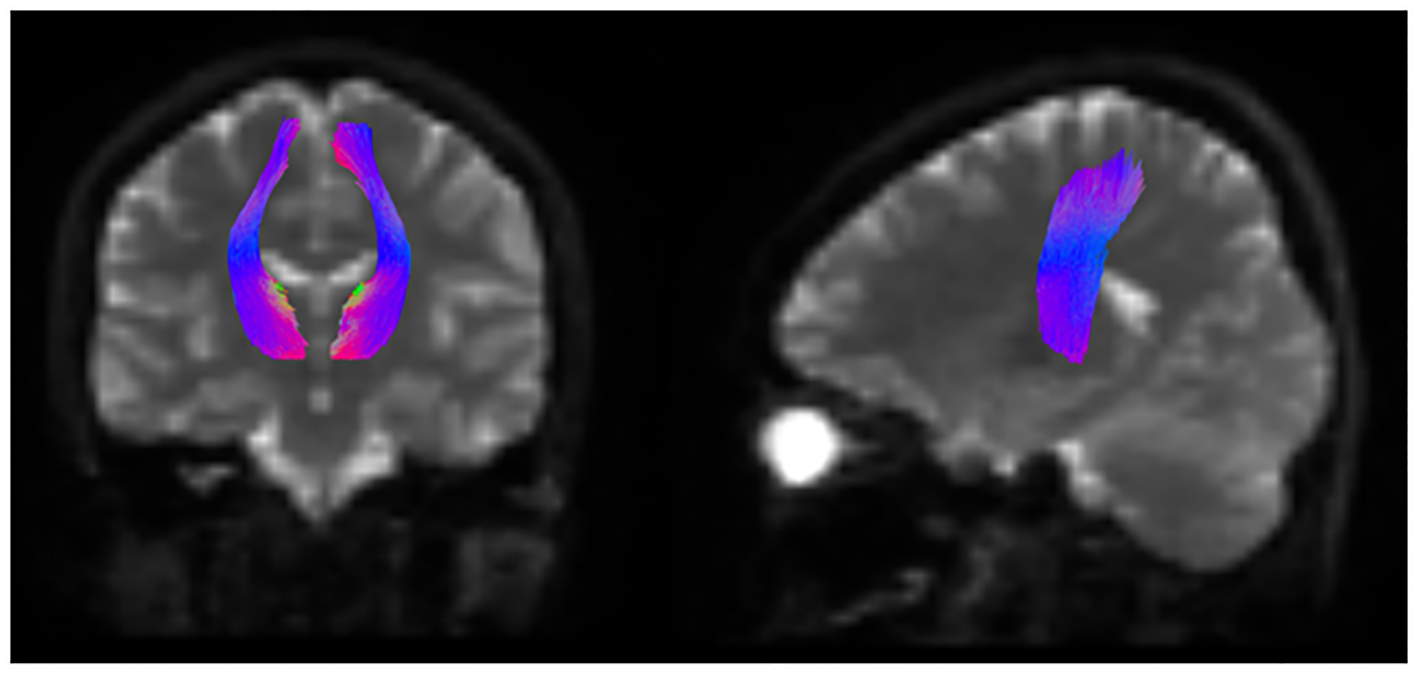
Schematic representation of the white matter tract segmentation in the STR. Schema of segmentation of the white matter tracts of STR using TractSeg. STR, superior thalamic radiation.

Segmentation of the white matter tracts was performed using TractSeg (32), which allows semi-automatic reconstruction of fiber bundles in the individual’s native space. In this study, we focused on the STR (**Figure 2**).

### 2.5 Statistical analysis

In the initial step, demographic comparisons were made using a t-test for age and a chi-square test for sex and dominant eye. In the second step, we conducted a comparison between the individuals with APS and healthy controls. We compared the FA values of the left and right STR and EEM parameters (NEF, TESL, MESL, and RSS) using analysis of covariance, adjusting for values that were significantly different between the APS and healthy control groups in the first step.

Finally, the relationships between FA values of bilateral STR and parameters of saccades were evaluated using correlation analysis with Pearson’s correlation coefficient in both groups. We ensured the normality of the saccade parameter and FA value distributions by confirming skewness values below 2 and kurtosis values below 4 for all items (39). Statistical significance was set at *p* < 0.05. The analysis was performed using SPSS for Windows (version 23.0; IBM Corp., Armonk, NY, USA).

## 3 Results

In this study, 21 participants were diagnosed with APS. Among them, 5 were males, and 16 were females, with a mean age of 21.4 (SD = 5.8) years. Among the 21 individuals with APS, 12 were receiving antipsychotic treatment, with a mean daily chlorpromazine-equivalent dosage of prescribed antipsychotics of 122.9 (SD = 112.5) mg (40). Additionally, we recruited 30 healthy controls (19 males and 11 females) from independent community sources.

The demographic data and SOPS scores are presented in **Table 1**. Notably, the t-test for age (*t* = -2.965, *p* = 0.004) and the chi-square test for sex (*χ2 =* 7.746, *p* = 0.005) both revealed statistically significant differences.

**Table 1:** The demographic data.

Characteristic	APS (SD)	HC (SD)	*t/χ2*	*p*
Participants (Male/Female)	21(5/16)	30(19/11)	7.746	0.005**
Age (years, mean)	21.4 (5.8)	25.9 (4.9)	-2.965	0.004**
Dominant eye (right/left)	11/10	22/8	2.375	0.12
Mean scores of SOPS items				
positive symptom (mean)	13.6 (2.4)	–		
Negative symptom (mean)	14.9 (6.4)	–		
Disorganized symptom (mean)	5.8 (3.2)	–		
General symptom (mean)	9.2 (3.6)	–		

Age is compared using Student’s t-test. Sex and dominant eyes are compared using the chi-square test.

SOPS, Scale of Prodromal Symptoms; SD, standard deviation; HC, healthy control group; APS, attenuated psychosis syndrome.

**p < 0.01.

The chi-square test for the dominant eye (χ2 = 2.375, p = 0.12) revealed no significant differences between the APS group and the healthy control group. The EEM parameters and FA values in the left and right STR are presented in **Table 2**. After adjusting for age and sex, the analysis of covariance revealed that the FA value of left STR (*F* [1, 47] = 5.713, *p* = 0.021), NEF (*F* [1, 47] = 4.419, *p* = 0.041), TESL (*F* [1, 47] = 4.967, *p* = 0.031) and MESL (*F* [1, 47] = 4.378, *p* = 0.042) were lower in the APS group compared to the healthy control group.

**Table 2:** Analyses of covariance with EEM parameters and FA value of STR.

	APS (SD)	HC (SD)	*F*	*p*
EEM Parameters				
NEF	28.3 (8.6)	32.6 (7.7)	4.419	0.041*
TESL (mm)	1286.0 (670.9)	1646.44 (725.6)	4.967	0.031*
MESL (mm)	43.6 (15.3)	49.7 (14.7)	4.378	0.042*
RSS	6.0 (2.7)	6.7 (2.6)	0.200	0.657
FA value				
STR_left	0.395 (0.021)	0.403 (0.020)	5.713	0.021*
STR_right	0.389 (0.018)	0.394 (0.016)	0.862	0.358

The FA values of the bilateral STR and EEM parameters are compared using an analysis of covariance that is adjusted for age and sex.

EEM, exploratory eye movement; NEF, number of eye fixations; TESL, total eye-scanning length; MESL, mean eye-scanning length; RSS, responsive search score; FA, fractional anisotropy; STR, superior thalamic radiation; SD, standard deviation; HC, healthy controls; APS, attenuated psychosis syndrome.

*p < 0.05.

In the APS group, Pearson’s correlation analysis revealed significant positive correlations between the FA values of the left STR and the MESL (*r* = 0.567, *p* = 0.007). Although the reduction in the FA value of the right STR was not significant in the APS group, significant correlations between the right STR and TESL (*r* = 0.587, *p* = 0.005) and MESL (*r* = 0.756, *p* = 0.7×10^-4^) were observed (**Table 3**). The SOPS items were not significantly correlated with EEM parameters or bilateral STR FA values (for detailed results, refer to **Supplementary Table 1**).

**Table 3:** Correlations between fractional anisotropy value of superior thalamic radiation and EEM parameters.

	Pearson	NEF	TESL	MESL	RSS
APS (n = 21)					
FA value of left STR	*r*	0.052	0.328	0.567	0.186
	*p*	0.823	0.146	0.007**	0.419
FA value of right STR	*r*	0.286	0.587	0.756	0.277
	*p*	0.209	0.005**	0.7×10^-4^**	0.223
HC (n = 30)					
FA value of left STR	*r*	0.301	0.278	0.141	-0.109
	*p*	0.107	0.137	0.459	0.568
FA value of right STR	*r*	-0.067	0.081	0.122	-0.426
	*p*	0.724	0.672	0.521	0.019*

EEM, exploratory eye movements; NEF, number of eye fixations; TESL, total eye scanning length; MESL, mean eye scanning length; RSS, responsive search score; FA, fractional anisotropy; STR, superior thalamic radiation; HC, healthy controls; APS, attenuated psychosis syndrome.

*p < 0.05. **p < 0.01.

## 4 Discussion

### 4.1 EEM parameters and STR changes in APS

Consistent with previous studies on schizophrenia, our study revealed significant reductions in NEF, TESL, and MESL in the APS group. These results are also consistent with those of Shido et al. (30), who reported a significant reduction in NEF and TESL in the APS group compared with the healthy control group. Notably, our study additionally revealed a significant reduction in MESL in the APS group, a finding that differs from Shido’s study (30). This difference may have resulted from using an analysis of covariance to compare the APS and healthy control groups while adjusting for age and sex.

MESL has been associated with attention (41), perceptual reasoning (29), and particularly negative symptoms in schizophrenia (6, 41–44). Negative symptoms, rather than positive symptoms, have been proposed to have a biological basis, including genetic factors (45–47). As MESL is calculated by dividing TESL by NEF, that is, MESL = TESL/NEF (37), a reduction in TESL can consequently lead to a decrease in MESL. A reduction in TESL in patients with schizophrenia (6), which is negatively correlated with the severity of negative symptoms, has also been reported (44). McAvoy et al. (2012) revealed the relationship between fixation and thalamocortical connectivity in humans (21). Zhang et al. (2021) reported lower NEF values in patients with schizophrenia compared to healthy controls, with a further reduction in patients with schizophrenia with pronounced negative symptoms (48).

The FA values of the left STR were significantly lower in the APS group than in healthy controls. The STR is a part of the thalamocortical pathway, which has been repeatedly reported in ARMS and schizophrenia (49). Carletti et al. (2012) reported changes in FA values in the left superior corona radiata in the ARMS (27). These studies corroborate the pathological changes on the left side of the white matter fiber bundles connecting the frontal cortex and thalamus. The decrease in FA values in the STR on the left side in the APS group in this study is consistent with the results of previous studies. In terms of laterality, cross-dominance, which is the crossover among the dominance of the left and right side of the eye, hand, and foot, is associated with treatment resistance in schizophrenia (50). This association indicates that cross-dominance as a biological background relates to differences in schizophrenia, such as prognosis and/or other symptoms. In this study, all participants were right-handed, while left-eye dominance (i.e., cross-dominance) was observed in 47.6% of the APS group and 26.7% of the healthy group. However, no significant differences in cross-dominance were observed between the two groups (χ2 = 2.375, p = 0.12). Unlike the previous study (50), the sample size was small, and the dominant foot was not identified in this study. These factors possibly affected the non-findings of cross-dominance in the APS group. Meanwhile, several recent studies revealed that APS does not necessarily transition to schizophrenia but various psychiatric disorders such as depression, bipolar disorder, and personality disorder (transdiagnostic psychiatry) (51). Thus, it is indicated that the biological backgrounds of APS are more heterogeneous, and it may not necessarily coincide with the biological trait of schizophrenia.

### 4.2 Relationship between changes of STR and fixations and scan path lengths

The thalamus plays a pivotal role in multiple brain networks responsible for processing sensory input, and it is intricately linked to various higher-order cognitive and emotional functions (52). Dysfunctions in the electrophysiological coordination of cortico-thalamic connections have been associated with cognitive impairments (53, 54). Aberrant neural connections between the thalamus and cortex have been repeatedly reported in schizophrenia (55). However, whether biological changes in corticothalamic disconnection underlie aberrant eye movement and subthreshold psychotic symptoms in APS remains unclear.

In this study, we hypothesized that biological changes in the STR might be associated with aberrant eye movements, including fixation and scan path length abnormalities, among individuals with APS. Our analysis aimed to explore the potential correlation between STR FA values and the NEF, TESL, and MESL values within both the APS and healthy control groups.

In the APS group, a significant reduction in the FA value in the left STR was observed compared with that in the healthy group, and the FA value of the left STR was significantly correlated with the MESL. Although no reduction in the FA value in the right STR was observed compared to that in the healthy group, significant correlations between the FA values of the right STR, TESL, and MESL were observed. Considering that TESL and MESL are parameters of the scanning length, our findings raise the possibility that impairment of the white matter fiber bundles of the STR causes aberrant scanning in APS. This study is the first to report an association between STR, fixation, and scan path length in an APS group.

Qiu et al. (2018) reported that the severity of hallucination was negatively correlated with RSS and grey matter volume of bilateral precentral gyri and left supplementary motor area in schizophrenia (56). This report suggests that biological changes in STR appear in schizophrenia and are involved in auditory hallucinations and abnormalities of exploratory eye movements because the STR originated from the precentral gyri and supplementary motor area.

Transition to schizophrenia is defined by the expression of prominent positive symptoms, and sub-threshold psychotic symptoms gradually develop before the onset of schizophrenia.

Our study shows reduced white matter integrity of STR and abnormalities in EEM in the APS group of those who reveal sub-threshold psychotic symptoms. Therefore, we considered that biological changes in the STR and abnormalities in the EEM precede prominent psychotic symptoms, such as positive and negative symptoms.

### 4.3 Thalamus-related anomalies as candidate mechanism-based biomarkers for psychosis

Regarding the pathogenesis of schizophrenia, mounting evidence shows that abnormal connections within several brain circuits, including the thalamus, progress during the prodromal period, resulting in worse functional outcomes and the emergence of psychosis (22).

Yao et al. (2019) reported a relationship between decreased FA values in the thalamus-FEF pathway and the severity of psychotic symptoms in schizophrenia (49). Additionally, abnormal thalamocortical structural connectivity has been reported during the prodromal phase (57). In this study, we unveiled an anomaly in the STR, a component of the thalamocortical pathway, which correlated with shortened scan path lengths, representing a parameter of visual search in APS.

From the viewpoint of whole-brain networks, thalamo-cortical networks are part of the cortico-striatal-thalamic circuits. Abnormalities in the cortico-striatal-thalamic circuit may cause impairments in self-relevance processing and induce aberrant salience, which is proposed to be the biological background of self-disorder, the core symptom of schizophrenia (58). Moreover, abnormal neural connections between the cortex and thalamus lead to a hyperdopaminergic state in the striatum, resulting in psychiatric symptoms (59, 60). This study demonstrates that the scan path length in APS is associated with STR impairment, a component of the cortico-striatal-thalamic circuit, in individuals with APS exhibiting subthreshold psychotic symptoms. Kojima et al. (2019) predicted that EEM is a parameter of a subjective disorder, which is a core symptom of schizophrenia (31).

Finally, our results indicate that the scan path length may be a potential parameter of STR alterations in APS. In other words, there is a possibility that the reduction of scan path lengths could be predictive of STR impairment, which is presumed to be a part of the cortico-thalamic pathway associated with several psychotic symptoms, including self-disorder, in individuals with APS.

### 4.4 Limitations

This study has some limitations. First, the use of antipsychotic medication by some participants may have influenced both white matter fiber integrity and eye movement test outcomes. A more comprehensive investigation of biological changes would necessitate an analysis involving individuals who are not receiving antipsychotic medication. Second, the use of a 1.5-T scanner in this study could potentially limit the precision of the MRI data. Future analyses utilizing images obtained from 3-T MRI scanners would be preferable to enhance data quality. Third, in this study, the demographic data of the individuals in the APS and healthy control groups were significantly different in age and sex, which may have influenced the comparison of the two groups. Although we adjusted for age and sex to minimize this effect, in the follow-up study, it would be desirable to compare groups that are matched in age and sex. Fourth, this was a cross-sectional study. In a follow-up study, it would be necessary to examine the differences in longitudinal changes in STR and/or eye movement between individuals who developed psychosis and those who did not in the APS group to elucidate whether and how these biological changes are related to the emergence of psychotic symptoms.

## 5 Conclusion

Abnormalities in fixation and scan-path lengths have been observed as specific features associated with schizophrenia. Investigating the underlying biological basis of visual search in schizophrenia can clarify the etiology of aberrant neural networks in the brain, which are implicated in the development of schizophrenia. However, whether the abnormality of fixations and scan path lengths emerge before the onset of schizophrenia and what contributes to the abnormality of visual search remain unclear. In this study, we identified significant correlations between biological changes in the STR, including neural connections between the thalamus and the FEF, and anomalies in fixation and scanning in the APS group. The STR is a crucial component of the frontal–striatal–thalamic circuit and is responsible for broad mental activity in humans. An impairment of this circuit is supposed to cause psychotic symptoms partly. Possibly, impairment of the STR is related to the biological background of subthreshold psychotic symptoms as well as oculomotor disturbances. Furthermore, our findings, which demonstrated that biological changes in the STR correlated negatively with MESL, reveal that MESL can serve as an indicator of STR alterations in individuals with APS.

## Data availability statement

The datasets presented in this article are not readily available because the Ethics Committee of Toho University Omori Medical Center has not authorized to provide any data. Requests to access the datasets should be directed to ktgrnoyk@med.toho-u.ac.jp.

## Ethics statement

The studies involving humans were approved by the Ethics Committee of Toho University Omori Medical Center (A19078). The studies were conducted in accordance with the local legislation and institutional requirements. Written informed consent for participation in this study was provided by the participants’ legal guardians/next of kin. Written informed consent was obtained from the individual(s) for the publication of any potentially identifiable images or data included in this article.

## Author contributions

YA: Writing – original draft, Methodology, Funding acquisition, Formal Analysis, Data curation, Conceptualization. NK: Writing – review & editing, Writing – original draft, Supervision, Methodology, Funding acquisition, Formal analysis, Data curation, Conceptualization. HT: Writing – review & editing, Methodology, Data curation. TU: Writing – review & editing, Methodology, Data curation. JS: Writing – review & editing, Methodology, Data curation. YS: Writing – review & editing, Methodology, Data curation. KK: Writing – review & editing, Software, Methodology. MH: Writing – review & editing, Supervision, Software, Methodology. MM: Writing – review & editing, Supervision, Conceptualization. TN: Writing – review & editing, Supervision, Conceptualization.
