# Supplementary figures and images for: Exploring the impact of biological alterations in the superior thalamic radiations on exploratory eye movements in attenuated psychosis syndrome
Source: Front Psychiatry. 2024 Jun 13;15:1323786. doi: 10.3389/fpsyt.2024.1323786 (PMC11210316; doi:10.3389/fpsyt.2024.1323786)

Supplementary Figure 1

Sample images of exploratory eye movement of a control subject.

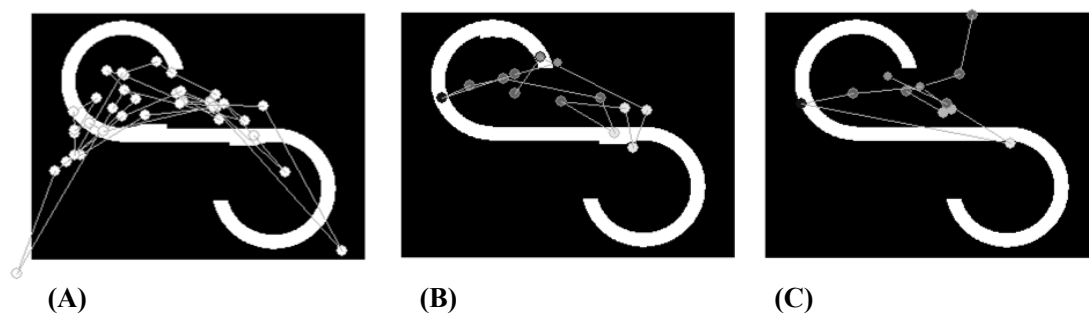

Supplement: Supplementary file 1 [file Image_1.pdf]
