# Supplementary material for: Exploring the impact of biological alterations in the superior thalamic radiations on exploratory eye movements in attenuated psychosis syndrome
Source: Front Psychiatry. 2024 Jun 13;15:1323786. doi: 10.3389/fpsyt.2024.1323786 (PMC11210316; doi:10.3389/fpsyt.2024.1323786)
Supplement: Supplementary file 2 [file Table_1.docx]

Supplementary Table 1. Correlations between Scale of Prodromal Symptoms and biological data in APS group

|  |  |  |  |  |  |  |  |
| --- | --- | --- | --- | --- | --- | --- | --- |
|  | Pearson's | NEF | TESL | MESL | RSS | FA value of left STR | FA value of right STR |
| APS (n = 21) |  |  |  |  |  |  |  |
| Scores of SOPS items |  |  |  |  |  |  |  |
| Positive symptoms | *r* | -0.176 | -0.203 | -0.104 | -0.010 | -0.297 | -0.185 |
|  | *p* | 0.444 | 0.379 | 0.655 | 0.964 | 0.192 | 0.422 |
| Negative symptoms | *r* | -0.214 | -0.114 | 0.039 | -0.139 | -0.272 | 0.090 |
|  | *p* | 0.350 | 0.624 | 0.865 | 0.549 | 0.234 | 0.697 |
| Disorganized symptoms | *r* | -0.364 | -0.245 | -0.055 | -0.365 | -0.370 | -0.05 |
|  | *p* | 0.105 | 0.284 | 0.813 | 0.103 | 0.099 | 0.829 |
| General symptoms | *r* | 0.034 | 0.088 | 0.084 | 0.035 | -0.188 | -0.026 |
|  | *p* | 0.883 | 0.706 | 0.716 | 0.88 | 0.415 | 0.910 |
|  |  |  |  |  |  |  |  |

SOPS, Scale of Prodromal Symptoms; EEM, exploratory eye movements; NEF, number of eye fixations; TESL, total eye scanning length; MESL, mean eye scanning length; RSS, responsive search score; FA, fractional anisotropy; STR, superior thalamic radiation; HC, healthy controls; APS, attenuated psychosis syndrome.
